# Linking root exudates to functional plant traits

**DOI:** 10.1371/journal.pone.0204128

**Published:** 2018-10-03

**Authors:** Katharina Herz, Sophie Dietz, Karin Gorzolka, Sylvia Haider, Ute Jandt, Dierk Scheel, Helge Bruelheide

**Affiliations:** 1 Martin Luther University Halle-Wittenberg, Institute of Biology / Geobotany and Botanical Garden, Halle (Saale), Germany; 2 Leibniz Institute of Plant Biochemistry, Halle (Saale), Germany; 3 German Centre for Integrative Biodiversity Research (iDiv) Halle-Jena-Leipzig, Leipzig, Germany; University of Leipzig, GERMANY

## Abstract

Primary and secondary metabolites exuded by plant roots have mainly been studied under laboratory conditions, while knowledge of root exudate patterns of plants growing in natural communities is very limited. Focusing on ten common European grassland plant species, we asked to which degree exuded metabolite compositions are specific to species or growth forms (forbs and grasses), depend on environments and local neighbourhoods, and reflect traditional plant functional traits. Root exudates were collected under field conditions and analysed using a non-targeted gas chromatography coupled mass spectrometry (GC-MS) approach. In total, we annotated 153 compounds of which 36 were identified by structure and name as metabolites mainly derived from the primary metabolism. Here we show by using variance partitioning, that the composition of exuded polar metabolites was mostly explained by plot identity, followed by plant species identity while plant species composition of the local neighbourhood played no role. Total and root dry biomass explained the largest proportion of variance in exudate composition, with additional variance explained by traditional plant traits. Although the exudate composition was quite similar between the two growth forms, we found some metabolites that occurred only in one of the two growth forms. Our study demonstrated the feasibility of measuring polar exudates under non-sterile field conditions by mass spectrometry, which opens new avenues of research for functional plant ecology.

## Introduction

Plant roots constantly exude compounds of the primary and secondary metabolism into the rhizosphere. Such exudates serve for nutrient acquisition and interaction of the plant with the root surrounding environment [1,2]. Their release is controlled and adjusted to the needs of a plant as well as to abiotic and biotic factors [3–5]. Until now, analyses of the composition of such metabolites were mostly done on model plants, such as *Arabidopsis thaliana*, for which up to 130 primary [6,3] and 103 secondary metabolites [7] have been described. These are sugars, sugar alcohols, phenolic compounds, organic acids, fatty acids as well as aliphatic and aromatic amino acids [6,3]. The functions described for those metabolites comprise (i) mobilization of soil nutrients with low availability, such as phosphorus; (ii) stimulation of external detoxification of metals; and (iii) mediation of positive and mutualistic interactions with beneficial, plant-growth promoting microorganisms, such as endophytic and rhizobial bacteria or mycorrhizal fungi [8–11].

In addition, the metabolic composition of exudates can be expected to be also affected by different neighbour plant species [10] via direct or indirect interaction with other plant roots [4]. Another potential factor influencing root exudation patterns might be the functional traits of the exuding plant individual. Plant functional traits have been found to be closely linked to individual plant performance [12–14]. For example, a high specific leaf area is linked to high relative growth rate [15], and thus might result in larger amounts of exuded carbon, as carbon exudation has been found to positively correlate with plant biomass [5]. Under nutrient deficiency more biomass is allocated to roots [16,17] which might be reflected by more exuded substances for nutrient mobilization [10,18]. Establishing links between exudate composition and plant traits would be very useful in ecological research since the impact of exudates in natural grasslands was underestimated so far. Combining morphological or anatomical traits with exudate pattern would allow for mechanistic explanations in ecosystem functioning which have been described so far for few selected substances only, but not for the overall exudate composition [5]. Furthermore, such analysis of causal relationships in the belowground ecosystem compartment is still heavily understudied [4] due to another challenge in this kind of investigation: the extraction of root exudates from field-grown plants under non-sterile conditions [4]. An ubiquitous problem is the damage of roots which can never be fully eliminated and which will alter the exudation profile [19]. However, Oburger and Jones stated that extraction of root exudates from field grown plants can still be used as a crude screening tool [19]. Here, we present a successful procedure to obtain root exudates from plants planted into natural grassland communities. Grassland communities consist of two main growth forms: grass and forbs. As the ecological functions of grass and forb species are distinct and they differ in several functional traits [20,21], we hypothesized 1) that these two growth forms differ in exudation patterns. Furthermore, we tested the hypotheses that root exudate composition of a target plant is correlated 2) with the plant’s functional traits and 3) depends on local neighbour plant species as well as conditions varying at the plot level.

## Methods

### Raising of the phytometer plants

The plants used for this experiment were raised under greenhouse conditions at the Botanical Garden Halle (Germany). Most seeds were collected in summer 2011 and 2013 in the three regions of the German Biodiversity Exploratories [22]. Additionally, to ensure a sufficient amount of seedlings, 500 seeds of 4 species (*Galium verum*, *Lolium perenne*, *Poa pratensis* and *Ranunculus acris*) were ordered from a local supplier for wild flowers and grasses (Rieger-Hofmann GmbH). The provenances of the ordered seeds were located near the Exploratory regions.

Before sowing took place in December 2013, seeds were sterilized with 70% ethanol for 30 sec, afterwards with 1% sodium hypochlorite for 1 min and finally washed three times with distilled water. Seeds were then sown on sterilized 1:1 sand and silt mixture. Germination and growing of the seedlings took place in the greenhouse until end of March 2014 at 20°C during daytime and 10°C during the night with a 12 h/12 h light/dark rhythm. The plants were watered with sterilised water every second day and fertilized three times in total (25.02.2014, 11.03.2014 and 25.03.2014) with Wuxal Super (MANNA, Wilhelm Haug GmbH & Co. KG, 8% N, 8% P2O5, 6% K2O, 0.01% B, 0.004% Cu, 0.02% Fe, 0.012% Mn, 0.001% Mo, 0.004% Zn). To allow for hardening, the plants were transferred to the outdoor area of the Botanical Garden Halle in April 2014. Seedlings were pooled and randomly selected for planting across the different seed origins.

### Experimental setup

The experiment took place during spring and summer 2014 in the three regions of the German Biodiversity Exploratories [22]: Schorfheide-Chorin, Hainich-Dün and Schwäbische Alb. Eighteen out of the 50 experimental grassland plots in each of the three Exploratories were selected varying in land use intensity, resulting in a total of 54 experimental plots, see also Herz et al. [20]. Each plot comprised an area of 7 x 11 m and included five blocks. Each block contained one individual (= one phytometer) of each of the total 20 study species. Of these, the present study used ten species: *Alopecurus pratensis* L., *Arrhenatherum elatius* (L.) P.Beauv. ex J.Presl & C.Presl., *Dactylis glomerata* L., *Lolium perenne* L., *Poa pratensis* L. (all Poaceae) and *Achillea millefolium* L. (Asteraceae), *Galium mollugo* L., *Galium verum* L. (Rubiaceae), *Plantago lanceolata* L. (Plantaginaceae), *Ranunculus acris* L. (Ranunculaceae). These perennial species are among the most frequent and abundant species in all plots of the Exploratories’ grasslands. Within blocks, the individuals of each species were planted in May and early June 2014 using random planting positions. The soil used for raising the plants was removed with tap water before planting to ensure a better adaptation of the plants to the local conditions. Each plant was marked with a plastic ring around the hypocotyl and a coloured bamboo stick to facilitate the recognition during the harvest. Three weeks after planting dead plants were replaced. Further replacements did not take place. An overview of the ten species and their sample sizes is given in S1 Table.

As access to some plots was restricted at the time of harvest and in some plots mortality was too high, we could not harvest the full set of planted phytometers. The phytometer species (five grasses and five forbs) were harvested in 46 of the 54 established plots (in total 304 individual plants) as follows in September 2014. We dug a hole of ca. 20 cm depth and 15 cm length and width to extract one individual, carefully removed the rhizosphere soil and roots of other plants and gently washed the roots with tap water. As the plants were only three months in the field by the time of harvest, the roots did not grow into other patches and could be easily extracted from the soil and surrounding plants. With this we are confident that it was possible to extract the phytometer plants completely without damaging their roots. However, we cannot fully exclude the possibility that some minor damage occurred when working under field conditions, which may be a potential source of error in our study. We measured several above- and belowground traits (S2 Table) as well as polar exuded metabolites (see below) on these phytometers.

Additionally, the composition of neighbour plant species at the location of each phytometer was obtained by recording the number of plant species and cover per species growing in a 15 cm radius (707 cm^2^) around each phytometer plant. These vegetation records were used to calculate species richness and Shannon diversity of neighbour plant species, in addition to species composition as obtained from the first four axes of a detrended correspondence analysis (DCA).

### Extraction of exudates

We successfully adapted a procedure developed by Aulakh et al. [5] to collect exudates directly form the roots in field. To reduce the content of ions from the tap water, we performed a second wash step of the roots with deionised water. Afterwards, the complete roots of the intact plant were placed in 250 ml brown plastic vessels (Nalgene) containing 200 ml of deionised water of HPLC quality for 2 hours exudation. Water was found to be the most effective extracting solution for collecting exudates also by Vallenrinuzzi et al. [23], who compared different trap solutions. Water samples of 200 ml deionised water of HPLC quality in brown plastic vessels (Nalgene) without exudation were used for process control (“water blanks”) and treated exactly like the exudate samples. All samples were frozen and stored at -20°C until further processing. After thawing the exudate solution, it was filtered (Sartorius; 185mm, 80g/cm^3^) and the water was successively evaporated under reduced pressure (30 mbar) at 40°C to dryness using a 100 ml round-bottom flask and a vacuum rotary evaporator. The extraction of metabolites was obtained by dissolving them two times in 3 ml 100% methanol (Sigma-Aldrich), sonicating them for 10 min at 20°C and then transferring the solution into 5 ml glass tubes (Agilent Technologies). The residuum was evaporated to dryness at 40°C using a vacuum centrifuge and reconstituted in 1.1 ml 80% MeOH containing 20 μg/mL 2- (2,4-dichlorophenoxy) acetic acid and 10 μM Ribitol as internal standards. An aliquot of 200 μl of each sample was centrifuged to precipitate remaining particles followed by the transfer of the supernatant to a new tube and drying in a vacuum concentrator. The metabolites in these samples were derivatized by methoxylamination with 50 μl methoxylamin-hydrochloride (20 mg/ml in pyridine, Sigma Aldrich) for 90 min at 37°C and subsequently silylated with 50 μl BSTFA (Macherey–Nagel) with added alkane retention time indices (C12, C15, C19, C22, C28 (each 0.1 mg/ml final concentration; Sigma Aldrich) and C32 (0.4 mg/ml final concentration); Sigma Aldrich)) for 30 min at 37°C [24].

### GC-MS analysis and data processing

Derivatized exudates and water controls were analysed by non-targeted plant metabolite profiling with gas chromatography coupled to mass spectrometry.

The measurements were performed using a gas chromatograph (6890N GC; Agilent Technologies) equipped with a ZB-5 Zebron Guardian^TM^ Capillary GC column (30 m + 10 m Zebron^TM^, iD 0.25 mm, df 0.25 μm; Phenomenex) and coupled to mass spectrometer (5975 MSD; Agilent Technologies) with settings and method adapted to Gorzolka et al. [24]. Samples (2 μl) were injected automatically by multipurpose sampler (MPS 2XL; Gerstel) at 230°C injector temperature and separated chromatographically with 1ml/min flow and the following oven program: 1 min 70°C, ramp with 7°C per minute up to 300°C, 5 min 300°C. The transfer line temperature was set at 300°C and ion source at 230°C. Mass spectra were recorded with 20 Hz with MS calibration obtained by daily automated tuning of the MS on PSTFA. Single samples were derivatized and measured separately with intervals of at least one day. A “chemical blank” (derivatization agents without biological sample) was interspersed every five to six samples to check for a potential carryover of metabolites during measurement. Constant chromatographic performance and sensitivity was checked by tune evaluation with PSTFA. The raw data were converted to cdf-files by the Data Analysis software (Agilent Technologies) and uploaded to the MeltDB software [25]. In MeltDB, peak detection with SN = 5 and FWHM = 6 using the warped-algorithm and metabolite profiling with threshold = 0.75 for compound conformation was done [25]. Identification of metabolites by mass spectra similarity was performed in MeltDB based on customized spectral and index libraries. Gaps in metabolite annotation were manually filled with the help of MeltDB, spectral and index libraries as well as Data Analysis (Agilent Technologies). Unidentified compounds were manually annotated by their mass to charge ratio (m/z) and retention time (RT). Metabolites and compounds occurring in 50% of water controls as well as in 50% of chemical blanks were regarded as artefacts and excluded from the metabolite list. Classification of metabolites was done according to their affiliation to natural substance classes.

### Ex-situ experiment on potential root damage by the exudate sampling procedure

To reveal the potential damage caused by our harvest and exudation procedure in the field we assembled a phyto-cabinet experiment which fully mimicked the field harvest procedure. The forb species *Plantago lanceolata* and the grass species *Arrhenatherum elatius* were grown from seeds under long daytime growth conditions (light phase: 16 hours, 150 μMol/m^2^s, 22°C, 60% humidity; dark phase: 8 hours, 0 μMol/m^2^s, 18°C, 60% humidity) in pots filled with a mixture of steam sterilized sand and silt (50:50) in phyto-cabinets for 56 days. After root cleaning and exudate collection analogous to the field experiment, dead and damaged cells were stained with lactophenol-trypanblue according to Koch et al. [26]. Afterwards one half of the vertically separated roots was immediately transferred into Trypan blue colour solution (0.1% Trypan blue dye (Sigma, Darmstadt, Germany) in one part of Lactophenol (25% (v/v) Glycerine, 25% (v/v) Lactic acid, 25% (v/v) Phenol (all Roth, Karlsruhe, Germany), 25% (v/v) tape water) and 2 parts of Ethanol (Roth, Karlsruhe, Germany)). To stain dead and damaged cells, roots were incubated for 3 hours under shaking at room temperature. Background staining of the root was removed by three to four washing steps with Chloral hydrate (2.5 g/mL, Sigma-Aldrich, Darmstadt, Germany). Whereas the first washing step was used to remove remaining colour solution, following washing steps where performed over night by shaking at room temperature. Afterwards, roots were washed two times with deionized distilled water and stored in glycerine-water solution (1:1 v/v) at 4°C until microscopy. Samples from each root were selected randomly, cut from the root system with a razor blade, transferred onto object slides (Menzel) with glycerine-water solution (1:1 v/v), covered with a coverslip (IDL) and subjected immediately to microscopic analysis.

### Microscopic analysis and image editing of the ex-situ experiment

Three independent bright-field microscopic images were recorded of three different regions (root tip and two different upper regions) of the selected stained root with an AZ 100 Multi-Purpose Zoom Microscope and the NIS Elements Imaging Software (both Nikon Instruments Inc., Melville, NY, USA). Overview images were conducted with an AZ-Plan Apo 1x (NA: 0.1/WD: 35 mm) objective with 5 x magnification. Furthermore, detailed images of root hairs were made using an AZ-Plan Apo 4x (NA: 0.4/WD: 20 mm) objective with 8 x magnification. Images were processed equally in contrast and brightness with the Gimp software (v. 2.8.14). The same program was used to adapt scale bar in color, font and position to scale bar automatically generated by the NIS Elements Imaging Software.

### Trait analyses

Root nutrient concentrations were obtained using at least 50 mg root powder for a digestion with nitric acid and analysing the samples with photometric phosphate assay (for P) and atomic absorption spectrometry (AAS vario 6; Analytik Jena, Germany) to obtain K, Ca and Mg. Samples with less than 50 mg root powder could not be used for the digestion and values of these samples were predicted by using near infrared spectroscopy (NIRS; OPUS version 7.0, Bruker Optics) similar to the methods used by Mir-Marqués et al. [27]. As all samples were subjected to NIRS, the spectra of samples which were also digested were used for calibration, allowing the predictions for samples with insufficient sample amount for digestion.

Root N and C content as well as C to N ratio were obtained from root powder using a C/N-analyser (vario EL cube; Elementar, Hanau, Germany). In total, the following above- and below-ground plant traits were measured on a total of the same 304 phytometers planted into 46 grassland communities (plots): specific leaf area (SLA), leaf area ratio (LAR), leaf and root dry matter content (LDMC, RDMC), root to shoot ratio (RSR), root volume (RVol), root mass per volume (RMV) as well as the dry mass of roots (DM roots), leaves (DM leaves), all aboveground organs (DM above) and the whole plant (DM total), root nutrient contents (C, N, P, K, Ca and Mg) and root C to N ratio (RCNR) (S2 Table). By using redundancy analysis (RDA), variance partitioning, procrustes analysis and principal component analysis (PCA), we identified the factors that accounted for variation in plant exudate composition of polar metabolites. Procrustes is used to compare the correlation of two ordinations by rotating and stretching the second ordination to fit maximally to the first ordination. The match is then tested with a permutation test. A more detailed description of the trait analysis is given in Herz et al. [20].

### Statistical analysis

Statistical analyses were performed with R (version 3.2.3 [28]). We carried out all analyses on exudates based on peak intensities and on presence/absence of metabolites. As the amount of single exudates varied strongly among samples, we standardized peak areas by an internal standard (Ribitol m/z = 217), log- or square-root-transformed them and scaled them by column (exudate compound) and/or row (sample). This still resulted in highly heterogeneous data that were driven by single exudates in single samples, when we were using the metabolite peak area as input value. Thus, we confined all analyses to the presence/absence of polar metabolites. In total, 35 samples of all traits were identified as outliers and excluded from the analyses. At first we excluded values of root P, K, Ca and Mg smaller than zero (wrong NIRS predictions) and values of RCC smaller than 1 as the latter were caused by an error in peak area estimation of the C/N-analyser. Then LDMC, SLA, LAR, RDMC, RSR, RVol, RMV, RNC, RKC, RCaC, DM roots, DM leaves, DM above and DM total were transformed by natural logarithm, RPC and RMgC by square root. To test for growth form-specificity of exudation patterns we conducted a redundancy analysis where the polar metabolites were tested against a presence/absence matrix of growth form (grass or forb). Furthermore, we used variance partitioning (varpart, package vegan [29]) to detect how much variance was explained by either target species identity, the plot identity, composition of neighbour plant species of each phytometer or the trait composition of the phytometer plant. To test for differences in variation between grasses and forbs we made the described variance partitioning analysis separately for the two growth forms. Finally, all exudates and traits (S2 Table) were subjected to a principal component analysis (PCA). The two PCAs of exudates and traits were then compared with a procrustes analysis (function protest, package vegan [29]) to assess the correlation between exudate and trait patterns. To check for spatial autocorrelation we calculated Moran’s I (Moran.I, package ape [30]) using the PCA of metabolites. We used the geographic distances between plots, as within plots the distance of plants was at minimum 0.5 m and at maximum 2.5 m, which we considered zero distance in Moran’s I calculation. We used the scores of the first and second axis of the PCA on metabolites as response variables and across all species.

## Results and discussion

We measured the root exudate composition of polar metabolites mainly derived from the primary metabolism and related it to 18 plant traits. To evaluate root damage caused by the exudate collection and the consequent potential contamination of exudates by root metabolites we performed trypan blue staining and microscopic analysis of representative species (grown in the phyto cabinet) that underwent the same sampling procedure as in the field (S1 File). The roots were intact and damage to root hairs was insignificant or very minor, which indicated that the measured metabolites were not released from damaged cells but were exuded. Although the applied method did not provide a quantitative value for root damage, it provides an indication of the root’s vitality. Thus, we are confident that our method of exudate collection has limited contamination from root damage to a minimum.

Out of the 153 compounds encountered by mass spectrometry, 36 could be identified and were classified into alcohols (4), amino acids (10), lipids (4), nucleic bases (1), nucleotides (1), organic acids (11) and sugars (5; S3 Table). To detect differences in exudate composition between grasses and forbs, a presence/absence matrix of these substances was subjected to a RDA with a presence/absence matrix of growth form as constraint.

**Fig 1:**
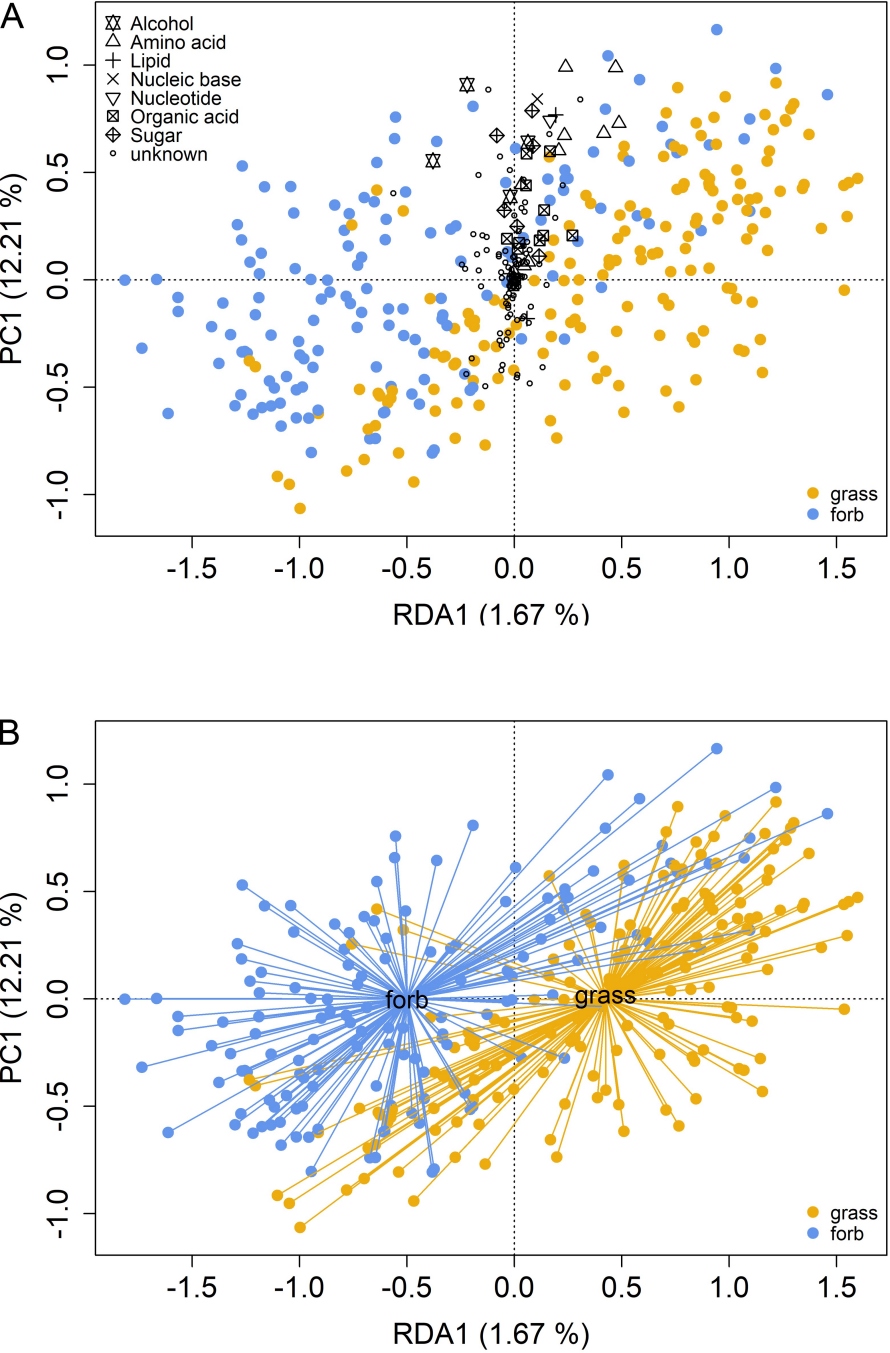
Redundancy analysis of polar metabolites and a presence/absence matrix of growth form. Symbols show compounds that could be attributed to the seven substance classes (see S3 Table) and unknown compounds (A). In part B points are grouped by growth form.

Grasses and forbs showed a largely common exudate composition which states the considerable low variation explained by the first RDA-axis (1.67%, Fig 1A and 1B). Nevertheless, grasses and forbs also showed differences in exudate patterns (Fig 1B) caused by many metabolites released predominantly by one of the two growth forms (S3 and S4 Tables), and thus confirming our first hypothesis. While for example adenosine and homoserine were released in more grass than forb samples, the opposite was true for e.g. scyllo-inositol and some unknown compounds (S4 Table, S1 Fig).

**Fig 2:**
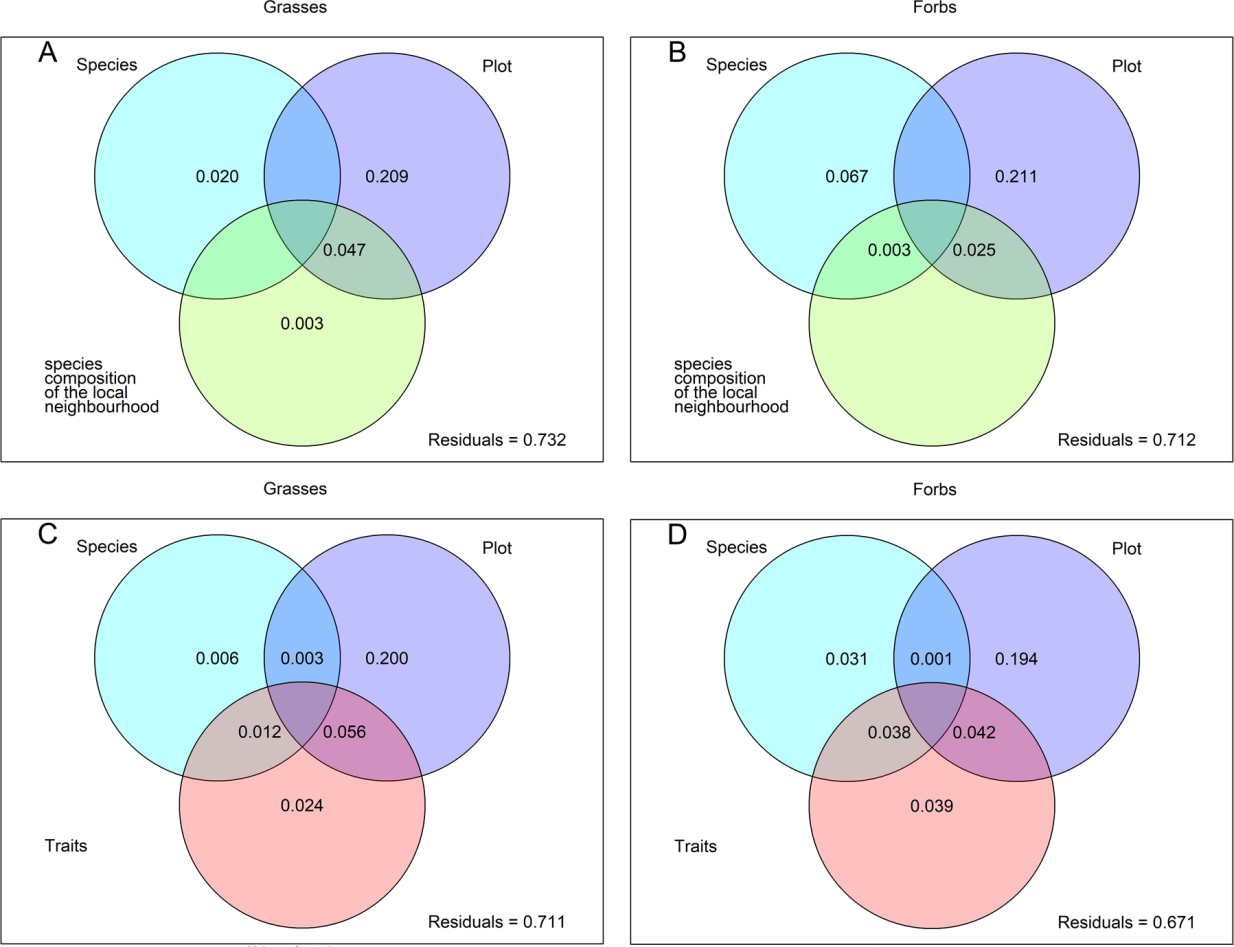
**Variance partitioning with proportion of explained variance of all polar metabolites of Fig 1 separately for grasses (A and C) and forbs (B and D).** Species = species identity of the phytometers, Plot = plot, where the phytometers were located between April and September 2014, species composition of the local neighbourhood = variables varying at the subplot level of 15 cm radius around the phytometer, including species richness, Shannon diversity, total cover and species composition of the local neighbourhood (obtained from the first four axes of a detrended correspondence analysis). The traits used as predictors in C and D are the same as in S2 Fig. Values below 0 are not shown.

To investigate which factors drive the variation in exudate composition in both growth forms, we used variance partitioning. In separate analyses for grasses and forbs, we used species identity, plot identity (which comprised all differences in ecological conditions between plots) and either species composition of the local plant neighbourhood or traits of the target plant as predictors for exudate composition. Similar to the results of Herz et al. [20] more variation was explained in forb species (28.8% to 32.9%, Fig 2B and 2D) than in grass species (26.8% to 28.9%, Fig 2A and 2C). Furthermore, interspecific variation of forbs was higher than that of grasses (Fig 2). This highlights the differences between these two growth forms and confirms the results of Herz et al. [20] for another important plant characteristic: exudate composition.

In addition, the variance partitioning showed that the exudation behaviour of both, grasses and forbs, is mainly driven by the plot into which they were planted (20.9% and 21.1%, respectively), which reflects the local environmental conditions. As the plots of the Exploratories are set up along a gradient of land use intensity (LUI), differences in exudation could also be linked to differences in LUI. However, in Herz et al. [20] we showed that edaphic and climatic conditions were more important for explaining variation in plant functional traits than was LUI, which also held true for variation in metabolites (analysis not shown). Species identity of the phytometer (2% in grasses and 6.7% in forbs) or species composition of the local neighbourhood in a 15 cm radius around the exuding plant (0.3% in grasses and 0% in forbs) contributed little to the total explained variation. The shared variation between plot and neighbouring plants of 4.7% in grasses and 2.5% in forbs points to a simultaneous account of effects of neighbour species and plot environment. This means that conditions varying at the plot level might be of greater importance for influencing root exudation of polar metabolites than the local plant neighbours or the species identity of the target plant. However, we cannot exclude that such species-specific adjustments to neighbour plants will not occur after a longer residence time of a plant like for example in the Jena experiment, where species richness effects on belowground organs were only visible four years after planting [31].

When we included phytometer traits instead of local plant neighbourhood composition the total amount of explained variation increased with traits explaining 2.4% in grasses and 3.9% in forbs (Fig 2). The identity of the plot still explained best the variation in plant exudation (20% in grasses and 19.4% in forbs), whereas species identity explained less than 4%.

**Fig 3:**
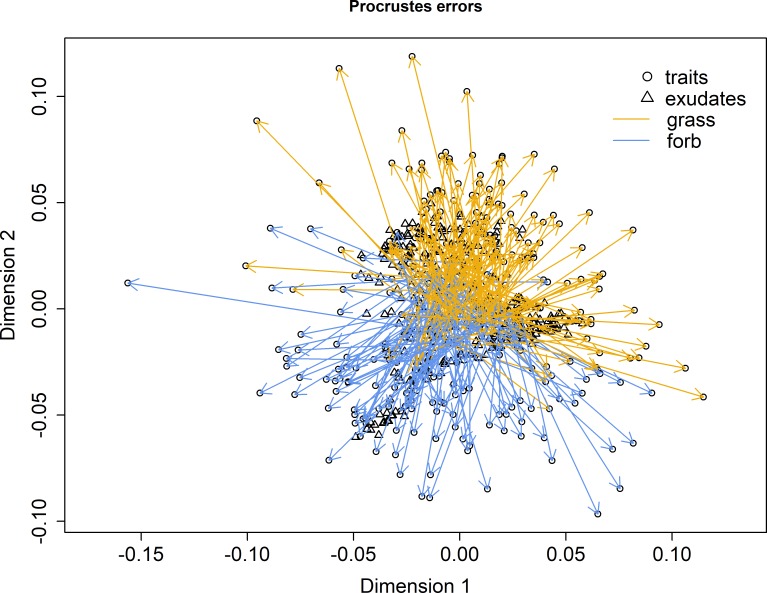
Procrustes correlation of S2 and S3 Figs. Arrows indicate in which direction the ordination is stretched to fit the ordination of traits to the ordination of the exudates. The two axes correspond to the principal components of the principal component analysis (PCA) of polar metabolites (S3 Fig). Correlation of the symmetric procrustes rotation = 0.2995, p = 0.001, number of permutations = 999.

We further compared the PCA of functional traits (S2 Fig) with the PCA of exudates (S3 Fig) in a procrustes analysis (Fig 3) to relate functional traits and exudates to each other. It revealed a high congruence between trait and exudate composition (R^2^ = 0.2995, p = 0.001) thus supporting our second hypothesis. By comparing the effects of each single trait on exudate composition using variance partitioning we revealed total and root dry mass as the most important traits (S2 and S3 Files) which pointed to shifts in exudate composition with plant biomass. This finding conforms to the reports that exudate composition of a plant changes with developmental stage and biomass [5]. For rice plants Aulakh et al. [5] reported an increase of exudation rates from the seedling stage till panicle initiation and a subsequent decrease at maturity. Therefore, young plants with low biomass can acquire more nutrients while adult plants with higher biomass invest more into storage. Furthermore, it was shown by Aulakh et al. that seedlings exuded a lower absolute amount of metabolites than adult plants [5]. However, the explained variation did not exceed 2.48% for any single trait included in our analysis and thus shows that exudate composition is not driven by one specific trait alone.

Overall we could show that exudates of plants growing in a natural community were much more determined by the environmental conditions of the plot rather than by species identity, by species-specific traits or neighbouring plants in this study. In contrast to our third hypothesis, root exudation patterns were almost unaffected by neighbour plant species composition. Thus the match between traits and exudates as shown in the procrustes analysis (Fig 3) was probably mainly driven by the environmental conditions of a plot that affected both traits and exudates. Nevertheless, to unravel the role of such environmental conditions as climate, soil properties, land use intensity or inclination on root exudation [32,21,33,34] is still a challenge. This is particularly true for our approach of analysing root exudate pattern on individual plants in natural communities as compared to root exudates at the level of artificially assembled communities in microcosms (see [35]). The challenge of analysing the drivers of root exudation also increases with the number of analysed compounds. While Eisenhauer et al. [35] measured a total of 15 exudates with HPLC, we annotated 153 compounds with mass spectrometry and included them in the analysis.

With our results we could already account for up to 32.9% of the total variation in exudate composition by the factors included in our study, although our phytometers have been exposed to their environment only for one vegetation season. The remaining proportion indicates that further variation in exudate composition might be explained by other factors such as seasonal variation, but also stochastic events within plots such as grazers’ trampling or defecating on single target plants or plot- and species-specific effects of the microbial community composition, herbivory, pathogens or endophytes. It would be of great interest to examine their influence on plant exudation behaviour, also for short and long time exposure in a natural environment.

Finally, plant exudate composition comprises not only the presented polar metabolites but also semi-polar metabolites characteristic of the secondary metabolism [4]. The technique of planting phytometers, retrieving them and collecting their root exudates could also allow for analysing such secondary metabolites, which is work in progress. Secondary metabolites have been found to be of considerable importance for plant interactions as they for example inhibit the growth of competing plant species [36] but also stimulate the germination of parasite seeds [37]. As root exudation depends on as yet unpredictable interactions in the rhizosphere, further integration of such metabolic data on root exudates with information on rhizosphere microbial community composition may fundamentally change our understanding of belowground interactions.

## Supporting information

S1 Table Number of samples from each of the ten study species. (PDF)

S2 Table List of used plant traits including abbreviations, category, unit and description. (PDF)

S3 Table List of identified polar metabolites of the gas chromatography coupled mass spectrometry approach. (PDF)

S4 Table List of all metabolites occurring in the two growth forms. (PDF)

S1 Fig Frequency of selected metabolites occurring in the two growth forms. (PDF)

S2 Fig Principal component analysis (PCA) of plant traits. (PDF)

S3 Fig Principal component analysis (PCA) of polar metabolites. (PDF)

S1 File Representative microscopic images of exuded plant roots stained by Trypan blue. (PDF)

S2 File Explained variance of exudate composition using single traits. (PDF)

S3 File Explained variance of exudate data in grasses and forbs using single traits. (PDF)
