# Correction: Linking root exudates to functional plant traits

**DOI:** 10.1371/journal.pone.0213965

**Published:** 2019-03-13

**Authors:** Katharina Herz, Sophie Dietz, Karin Gorzolka, Sylvia Haider, Ute Jandt, Dierk Scheel, Helge Bruelheide

The Data Availability statement for this paper includes an outdated link for access to the exudate data stored in the MetaboLights repository. The correct, complete Data Availability statement with updated MetaboLights repository information is: The minimal data set for this study has been uploaded to public repositories. The trait data is stored in the TRY Plant Trait Database: DOI: 10.17871/TRY.18 and the exudate data is stored in MetaboLights: https://www.ebi.ac.uk/metabolights/MTBLS687.
