# Supplementary material for: Linking root exudates to functional plant traits
Source: PLoS One. 2018 Oct 3;13(10):e0204128. doi: 10.1371/journal.pone.0204128 (PMC6169879; doi:10.1371/journal.pone.0204128)
Supplement: S1 Table — (PDF) [file pone.0204128.s001.pdf]

S1 Table. Overview of the ten study species. No. individuals show the number of individuals which could be analysed in this study. In total 304 phytometer individuals were analysed, 164 grasses and 140 forbs.

Growth form	Family	Species	No. individuals
Grass	*Poaceacea*	*Alopecurus pratensis* L.	37
Grass	*Poaceacea*	*Arrhenatherum elatius* (L.) P.Beauv. ex J.Presl & C.Presl.	29
Grass	*Poaceacea*	*Dactylis glomerata* L.	39
Grass	*Poaceacea*	*Lolium perenne* L.	30
Grass	*Poaceacea*	*Poa pratensis* L.	29
Forb	*Asteraceae*	*Achillea millefolium* L.	29
Forb	*Rubiaceae*	*Galium mollugo* L.	33
Forb	*Rubiaceae*	*Galium verum* L.	27
Forb	*Plantaginaceae*	*Plantago lanceolata* L.	29
Forb	*Ranunculaceae*	*Ranunculus acris* L.	22
