# Supplementary material for: Linking root exudates to functional plant traits
Source: PLoS One. 2018 Oct 3;13(10):e0204128. doi: 10.1371/journal.pone.0204128 (PMC6169879; doi:10.1371/journal.pone.0204128)
Supplement: S2 Table — (PDF) [file pone.0204128.s002.pdf]

S2 Table. List of used plant traits including abbreviations, category, unit and description.

Trait	Abbreviation	Unit	Description
Leaf dry matter content	LDMC	mg/g	Leaf dry mass per leaf fresh mass
Specific leaf area	SLA	m^2/kg	Leaf area per leaf dry mass
Leaf area ratio	LAR	cm^2/g	Leaf area per total dry mass
Root dry matter content	RDMC	mg/g	Root dry mass per root fresh mass
Root to shoot ratio	RSR		Root dry mass per aboveground dry mass
Root volume	RVol	cm^3	Root volume
Root mass per volume	RMV	g/cm^3	Root dry mass per scanned root volume
Root carbon content	RCC	%	Root carbon content
Root nitrogen content	RNC	%	Root nitrogen content
Root carbon to nitrogen ratio	RCNR		Root carbon to nitrogen ratio
Root phosphorus content	RPC	µmol/g	Root phosphorus content
Root potassium content	RKC	µmol/g	Root potassium content
Root magnesium content	RMgC	µmol/g	Root magnesium content
Root calcium content	RCaC	µmol/g	Root calcium content
Root dry mass	DM roots	g	Root dry mass
Leaf dry mass	DM leaves	g	Leaf dry mass
Aboveground dry mass	DM above	g	including shoots, leaves and flowers
Total dry mass	DM total	g	Dry mass of whole plant
