# Supplementary material for: Linking root exudates to functional plant traits
Source: PLoS One. 2018 Oct 3;13(10):e0204128. doi: 10.1371/journal.pone.0204128 (PMC6169879; doi:10.1371/journal.pone.0204128)
Supplement: S3 Table — (PDF) [file pone.0204128.s003.pdf]

S3 Table. **List of identified polar metabolites of the gas chromatography coupled mass spectrometry approach.** Columns show the substance, the affiliation to natural substance classes, the mass of quantification, the corresponding retention index and the number of plant samples per growth form in which the metabolites occur, out of a total of 164 and 140 for grass and forb species, respectively.

Metabolite	Metabolite class	Quantification mass [m/z]	Retention index [RI]	No. of grass phytometers	No. of forb phytometers
Pinitol (260)	Alcohol	260	1868	120	97
scyllo-inositol (204)	Alcohol	204	2072	107	124
scyllo-inositol (305)	Alcohol	305	1995	73	64
Xylitol (307)	Alcohol	307	1735	82	89
Arginine (256)	Amino acid	256	1843	7	2
Asparagine (245)	Amino acid	245	1697	16	8
beta-Alanine (248)	Amino acid	248	1473	105	49
Glutamine (155)	Amino acid	155	1485	1	1
Homoserine (218)	Amino acid	218	1463	49	24
Lysine (156)	Amino acid	156	1939	28	21
Methionine (176)	Amino acid	176	1532	69	23
Ornithine / Citrullin (142)	Amino acid	142	1840	85	52
Tryptophan (202)	Amino acid	202	2250	47	20
Tyrosine (218)	Amino acid	218	1960	116	57
Digalactosylglycerol (204)	Lipid	204	3218	143	117
Octadecadienoic acid (337)	Lipid	337	2218	71	44
Octadecatrienoic acid (335)	Lipid	335	2230	1	2
sn-Glycerol-3-phosphate (357)	Lipid	357	1797	47	33
Adenine (264)	Nuclic base	264	1883	101	77
Adenosine (236)	Nucliotide	236	2679	107	77
2-Aminoadipate (260)	Organic acid	260	1739	32	13
2-Isopropylmalate (275)	Organic acid	275	1600	7	4
3-Caffeoyl-trans-quinic acid (345)	Organic acid	345	3166	2	2

Metabolite	Metabolite class	Quantification mass [m/z]	Retention index [RI]	No. of grass phytometers	No. of forb phytometers
4-Aminobutanoate [GABA] (174)	Organic acid	174	1543	157	122
Aminomalonic acid (218)	Organic acid	218	1483	26	12
Gluconate (333)	Organic acid	333	2049	157	137
Phosphoenol-pyruvate (247)	Organic acid	247	1623	62	48
Ribonic_acid-gamma-lactone_like (204) RT1888	Organic acid	204	2784	43	32
Salicylic acid (267)	Organic acid	267	1533	11	8
Shikimate (204)	Organic acid	204	1841	153	119
Syringic acid (342)	Organic acid	342	1912	66	33
Glucose 6-phosphate (387)	Sugar	387	2393	14	2
Lactose (361)	Sugar	361	2734	48	48
Melibiose (361)	Sugar	361	2946	140	112
Myo-Inositol-1-phosphate (318)	Sugar	318	2487	13	10
Rhamnose (117)	Sugar	117	1756	130	115
