# Supplementary material for: Linking root exudates to functional plant traits
Source: PLoS One. 2018 Oct 3;13(10):e0204128. doi: 10.1371/journal.pone.0204128 (PMC6169879; doi:10.1371/journal.pone.0204128)
Supplement: S4 Table — (PDF) [file pone.0204128.s004.pdf]

**S4 Table. List of all metabolites occurring in the two growth forms.** Values (in percent) for grasses and forbs show in how many samples of these two growth forms all detected metabolites occur. Metabolites with a difference of more than 5 % are marked with an asterisk and presented in the bar plot in S1 Fig.

| Metabolite | Growth form | | Difference | |
|---|---|---|---|---|
| | grass | forb | | |
| 2-Aminoadipate (260) | 19.512 | 13.571 | 5.941 | * |
| 2-Isopropylmalate (275) | 4.268 | 3.571 | 0.697 | |
| 3-Caffeoyl-trans-Quinic acid (345) | 1.220 | 2.857 | 1.638 | |
| 4-Aminobutanoate [GABA] (174) | 95.732 | 92.143 | 3.589 | |
| Adenine (264) | 61.585 | 57.143 | 4.443 | |
| Adenosine (236) | 65.244 | 59.286 | 5.958 | * |
| Aminomalonic acid (218) | 15.854 | 12.857 | 2.997 | |
| Arginine (256) | 4.268 | 3.571 | 0.697 | |
| Asparagine (245) | 9.756 | 7.143 | 2.613 | |
| beta-Alanine (248) | 64.024 | 49.286 | 14.739 | * |
| Digalactosylglycerol (204) | 87.195 | 87.857 | 0.662 | |
| Gal_spec unknown (204) RT1888_Ribonic_acilactone | 26.220 | 23.571 | 2.648 | |
| Gluconate (333) | 95.732 | 95.000 | 0.732 | |
| Glucose-6-phosphate (387) | 8.537 | 5.000 | 3.537 | |
| Glutamine (155) | 0.610 | 1.429 | 0.819 | |
| sn-Glycerol-3-phosphate (357) | 28.659 | 20.714 | 7.944 | * |
| Homoserine (218) | 29.878 | 20.000 | 9.878 | * |
| Lactose (361) | 29.268 | 30.714 | 1.446 | |
| Melibiose (361) | 85.366 | 80.714 | 4.652 | |
| Lysine (156) | 17.073 | 14.286 | 2.787 | |
| Methionine (176) | 42.073 | 28.571 | 13.502 | * |
| Myo-Inositol-1-phosphate (318) | 7.927 | 7.143 | 0.784 | |
| Octadecadienoic acid (337) | 43.293 | 35.000 | 8.293 | * |
| Octadecatrienoic acid (335) | 0.610 | 0.714 | 0.105 | |
| Ornithine / Citrullin (142) | 51.829 | 42.143 | 9.686 | * |
| Phosphoenolpyruvate (247) | 37.805 | 32.143 | 5.662 | * |
| Pinitol (260) | 73.171 | 73.571 | 0.401 | |
| Rhamnose (117) | 79.268 | 79.286 | 0.017 | |

| Metabolite | Growth form | | Difference | |
|---|---|---|---|---|
| | grass | forb | | |
| scyllo-inositol (305) | 44.512 | 47.143 | 2.631 | |
| Shikimate (204) | 93.293 | 92.857 | 0.436 | |
| Syringic acid (342) | 40.244 | 40.000 | 0.244 | |
| Tryptophan (202) | 28.659 | 19.286 | 9.373 | * |
| Tyrosine (218) | 70.732 | 55.000 | 15.732 | * |
| Xylitol (307) | 50.000 | 54.286 | 4.286 | |
| unknown compound (184) RT704 | 0.610 | 0.714 | 0.105 | |
| unknown compound (261) RT1755 | 7.317 | 9.286 | 1.969 | |
| unknown compound (306) RT832 | 4.268 | 7.143 | 2.875 | |
| unknown compound (349) RT1833 | 6.098 | 7.857 | 1.760 | |
| unknown compound (217) RT1622 | 18.902 | 10.714 | 8.188 | * |
| unknown sugar (319) RT1278 | 0.000 | 2.143 | 2.143 | |
| unknown sugar (319) RT1481 | 2.439 | 2.143 | 0.296 | |
| unknown sugar (319) RT1485 | 68.293 | 69.286 | 0.993 | |
| unknown sugar (204) RT1613 | 91.463 | 92.857 | 1.394 | |
| unknown sugar (204) RT1781 | 3.049 | 8.571 | 5.523 | * |
| unknown sugar (261) RT1789 | 3.659 | 7.143 | 3.484 | |
| unknown sugar (204) RT1795/1798 | 18.902 | 29.286 | 10.383 | * |
| unknown sugar (361) RT1972, Melibiose | 0.000 | 4.286 | 4.286 | |
| unknown compound (214) RT436_2 | 19.512 | 17.143 | 2.369 | |
| unknown compound (214) RT437 | 17.073 | 15.000 | 2.073 | |
| unknown compound (175) RT438 | 1.220 | 4.286 | 3.066 | |
| unknown compound (153) RT445 | 21.341 | 23.571 | 2.230 | |
| unknown compound (153) RT445_2 | 0.610 | 0.000 | 0.610 | |

| Metabolite | Growth form | | Difference | |
|---|---|---|---|---|
| | grass | forb | | |
| unknown compound (174) RT467 | 46.341 | 54.286 | 7.944 | * |
| unknown compound (75) RT468 | 1.220 | 2.143 | 0.923 | |
| unknown compound (71) RT483 | 31.098 | 31.429 | 0.331 | |
| unknown compound (130) RT503 | 1.220 | 0.000 | 1.220 | |
| unknown compound (138) RT511 | 2.439 | 2.857 | 0.418 | |
| unknown compound (133) RT514 | 1.829 | 2.857 | 1.028 | |
| unknown compound (355) RT528 | 9.146 | 8.571 | 0.575 | |
| unknown compound (187) RT536 | 21.341 | 26.429 | 5.087 | * |
| unknown compound (177) RT541 | 2.439 | 2.857 | 0.418 | |
| unknown compound (188) RT544 | 32.317 | 34.286 | 1.969 | |
| unknown compound (281) RT550 | 0.610 | 1.429 | 0.819 | |
| unknown compound (369) RT550 | 2.439 | 2.857 | 0.418 | |
| unknown compound (132) RT561 | 25.000 | 30.714 | 5.714 | * |
| unknown compound (169) RT599 | 32.317 | 35.000 | 2.683 | |
| unknown compound (266) RT602 | 77.439 | 76.429 | 1.010 | |
| unknown compound (281) RT602 | 0.610 | 0.714 | 0.105 | |
| unknown compound (369) RT609 | 18.293 | 18.571 | 0.279 | |
| unknown compound (211) RT629_2 | 9.756 | 7.857 | 1.899 | |
| unknown compound (341) RT696 | 2.439 | 2.143 | 0.296 | |
| unknown compound (239) RT722 | 38.415 | 32.857 | 5.557 | * |
| unknown compound (239) RT723 | 0.610 | 1.429 | 0.819 | |

| Metabolite | Growth form | | Difference | |
|---|---|---|---|---|
| | grass | forb | | |
| unknown compound (234) RT730 | 31.707 | 30.714 | 0.993 | |
| unknown compound (156) RT781 | 44.512 | 42.143 | 2.369 | |
| unknown compound (116) RT783 | 0.610 | 0.714 | 0.105 | |
| unknown compound (261) RT784 | 4.878 | 2.857 | 2.021 | |
| unknown compound (213) RT790 | 3.659 | 2.143 | 1.516 | |
| unknown compound (234) RT801 | 12.805 | 8.571 | 4.233 | |
| unknown compound (188) RT808 | 31.707 | 40.714 | 9.007 | * |
| unknown compound (160) RT809 | 4.878 | 5.000 | 0.122 | |
| unknown compound (188) RT837 | 0.000 | 0.000 | 0.000 | |
| unknown compound (143) RT854 | 1.220 | 0.714 | 0.505 | |
| unknown compound (188) RT854 | 0.610 | 0.714 | 0.105 | |
| unknown compound (255) RT855 | 0.000 | 0.714 | 0.714 | |
| unknown compound (247) RT865 | 0.610 | 0.714 | 0.105 | |
| unknown compound (231) RT866 | 1.829 | 0.714 | 1.115 | |
| unknown compound (247) RT866_2 | 11.585 | 8.571 | 3.014 | |
| unknown compound (174) RT886 | 0.000 | 0.000 | 0.000 | |
| unknown compound (174) RT886_2 | 4.268 | 7.857 | 3.589 | |
| unknown compound (174) RT887 | 2.439 | 2.143 | 0.296 | |
| unknown compound (239) RT936 | 16.463 | 15.000 | 1.463 | |
| unknown compound (227) RT943 | 24.390 | 23.571 | 0.819 | |
| unknown compound (129) RT968 | 1.220 | 0.000 | 1.220 | |

| Metabolite | Growth form | | Difference | |
|---|---|---|---|---|
| | grass | forb | | |
| unknown compound (245) RT986 | 64.634 | 58.571 | 6.063 | * |
| unknown compound (223) RT987 | 4.268 | 5.714 | 1.446 | |
| unknown compound (245) RT987 | 23.780 | 23.571 | 0.209 | |
| unknown compound (262) RT1020 | 0.610 | 5.000 | 4.390 | |
| unknown compound (158) RT1048 | 3.049 | 5.714 | 2.666 | |
| unknown compound (245) RT1048 | 1.829 | 2.857 | 1.028 | |
| unknown compound (260) RT1063 | 3.049 | 5.714 | 2.666 | |
| unknown compound (274) RT1114 | 9.146 | 6.429 | 2.718 | |
| unknown compound (221) RT1115 | 0.610 | 0.714 | 0.105 | |
| unknown compound (103) RT1120 | 0.000 | 0.000 | 0.000 | |
| unknown compound (271) RT1139 | 9.146 | 7.143 | 2.003 | |
| unknown compound (174) RT1141_2 | 53.659 | 48.571 | 5.087 | * |
| unknown compound (174) RT1142 | 7.927 | 5.000 | 2.927 | |
| unknown compound (217) RT1161 | 0.610 | 0.000 | 0.610 | |
| unknown compound (193) RT1196 | 3.659 | 2.143 | 1.516 | |
| unknown compound (174) RT1210_2 | 3.049 | 3.571 | 0.523 | |
| unknown compound (174) RT1211 | 3.049 | 0.714 | 2.334 | |
| unknown compound (286) RT1213 | 3.049 | 5.714 | 2.666 | |
| unknown compound (374) RT1240 | 1.220 | 1.429 | 0.209 | |
| unknown compound (330) RT1241 | 7.927 | 5.714 | 2.213 | |
| unknown compound (345) RT1250 | 21.951 | 28.571 | 6.620 | * |

| Metabolite | Growth form | | Difference | |
|---|---|---|---|---|
| | grass | forb | | |
| unknown compound (299) RT1308_2 | 0.000 | 0.714 | 0.714 | |
| unknown compound (299) RT1308 | 7.317 | 9.286 | 1.969 | |
| unknown compound (204) RT1389 | 2.439 | 2.857 | 0.418 | |
| unknown compound (82) RT1481 | 2.439 | 3.571 | 1.132 | |
| unknown compound (82) RT1486 | 0.000 | 0.000 | 0.000 | |
| unknown compound (167) RT1517 | 4.268 | 5.714 | 1.446 | |
| unknown compound (148) RT1541 | 2.439 | 2.857 | 0.418 | |
| unknown compound (148) RT1541_2 | 1.220 | 2.143 | 0.923 | |
| unknown compound (129) RT1551 | 0.610 | 1.429 | 0.819 | |
| unknown compound (82) RT1558 | 3.659 | 2.857 | 0.801 | |
| unknown compound (357) RT1570 | 0.610 | 1.429 | 0.819 | |
| unknown compound (285) RT1632 | 2.439 | 2.143 | 0.296 | |
| unknown compound (287) RT1632 | 1.220 | 0.714 | 0.505 | |
| unknown compound (167) RT1639 | 3.659 | 5.714 | 2.056 | |
| unknown compound (239) RT1642 | 4.878 | 2.857 | 2.021 | |
| unknown compound (217) RT1655 | 92.073 | 92.143 | 0.070 | |
| unknown compound (297) RT1661 | 1.829 | 1.429 | 0.401 | |
| unknown compound (82) RT1702 | 15.854 | 10.000 | 5.854 | * |
| unknown compound (97) RT1714 | 14.024 | 13.571 | 0.453 | |
| unknown compound (56) RT1715 | 0.610 | 1.429 | 0.819 | |
| unknown compound (199) RT1720 | 0.000 | 0.714 | 0.714 | |

| Metabolite | Growth form | | Difference |
|---|---|---|---|
| | grass | forb | |
| unknown compound (55) RT1748 | 0.610 | 0.714 | 0.105 |
| unknown compound (149) RT1786 | 4.268 | 5.714 | 1.446 |
| unknown compound (85) RT1847 | 2.439 | 2.857 | 0.418 |
| unknown compound (85) RT1847_2 | 1.829 | 2.857 | 1.028 |
| unknown compound (361) RT1927 | 0.000 | 0.714 | 0.714 |
| unknown compound (204) RT1934 | 1.829 | 1.429 | 0.401 |
