# Supplementary material for: Linking root exudates to functional plant traits
Source: PLoS One. 2018 Oct 3;13(10):e0204128. doi: 10.1371/journal.pone.0204128 (PMC6169879; doi:10.1371/journal.pone.0204128)
Supplement: S1 Fig — (PDF) [file pone.0204128.s005.pdf]

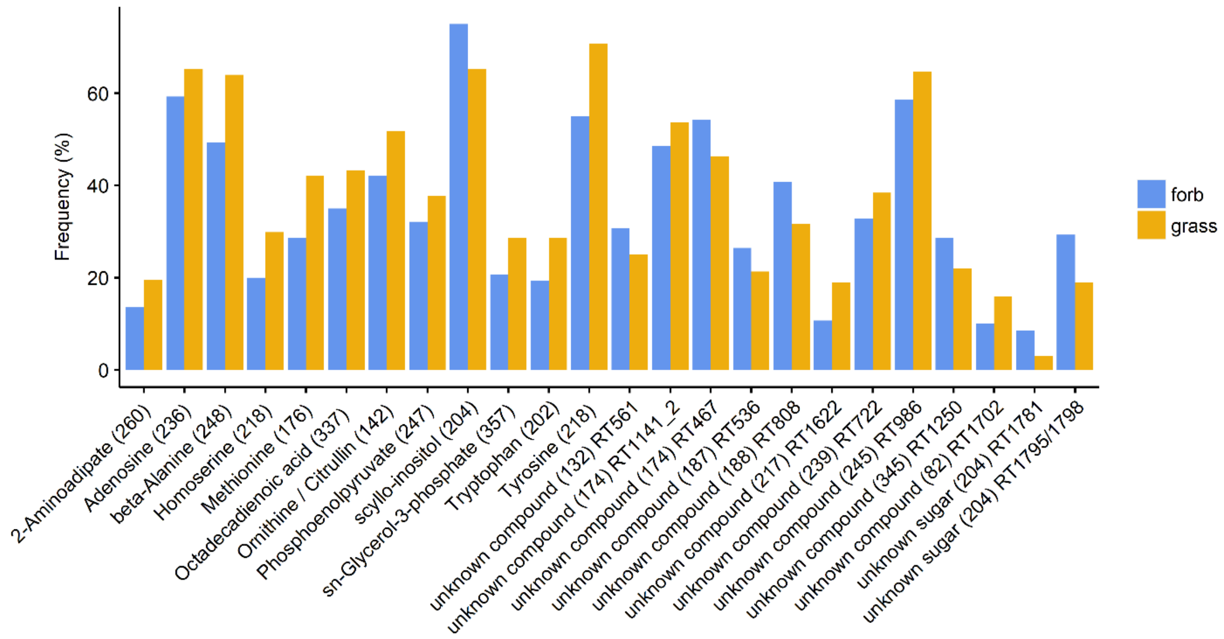


**S1 Fig. Frequency of selected metabolites occurring in the two growth forms.**

The values present the number of samples of forbs or grasses, respectively, in which a specific metabolite was detected, divided by the total number of analysed samples per growth form. Thereby, the graph presents all metabolites with a difference of more than 5 % in total occurrence between the two growth forms.
