# Supplementary material for: Linking root exudates to functional plant traits
Source: PLoS One. 2018 Oct 3;13(10):e0204128. doi: 10.1371/journal.pone.0204128 (PMC6169879; doi:10.1371/journal.pone.0204128)
Supplement: S2 Fig — (PDF) [file pone.0204128.s006.pdf]

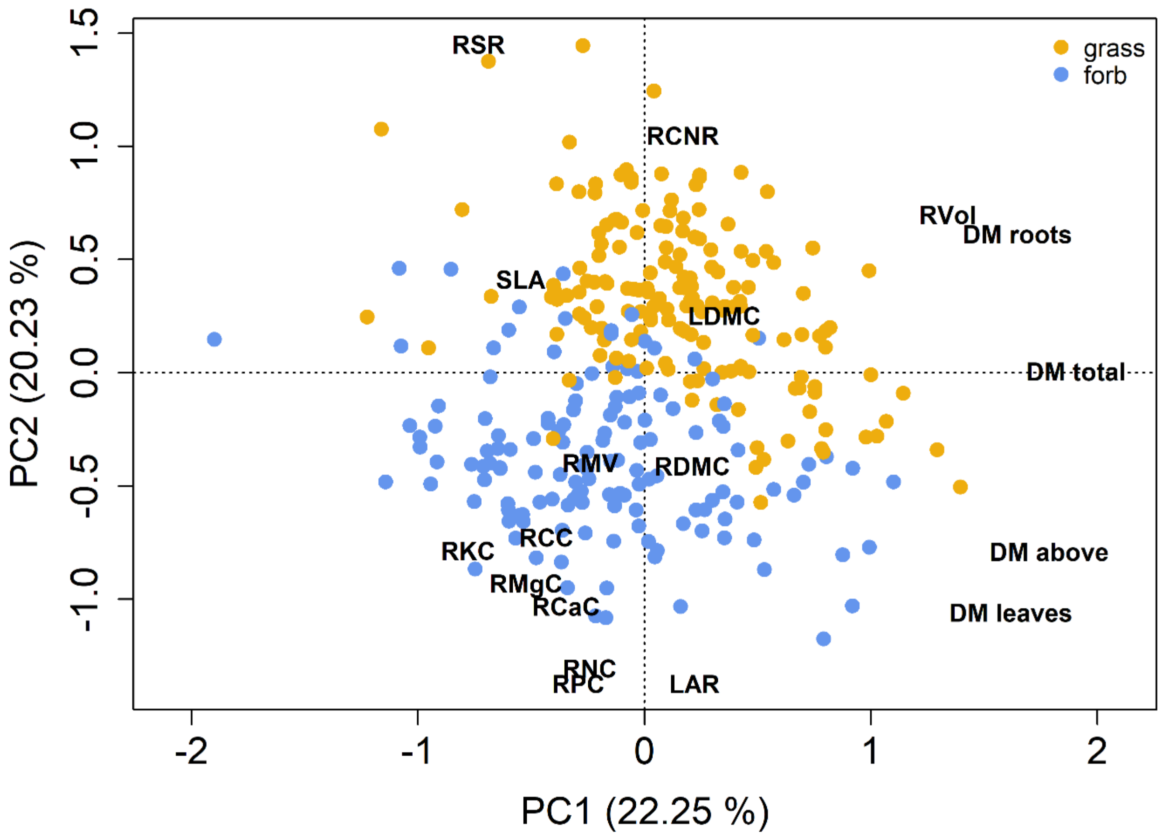

S2 Fig. Principal component analysis (PCA) of plant traits. For abbreviations see S2 Table. Colours represent the two growth forms.
