# Supplementary material for: Linking root exudates to functional plant traits
Source: PLoS One. 2018 Oct 3;13(10):e0204128. doi: 10.1371/journal.pone.0204128 (PMC6169879; doi:10.1371/journal.pone.0204128)
Supplement: S3 Fig — (PDF) [file pone.0204128.s007.pdf]

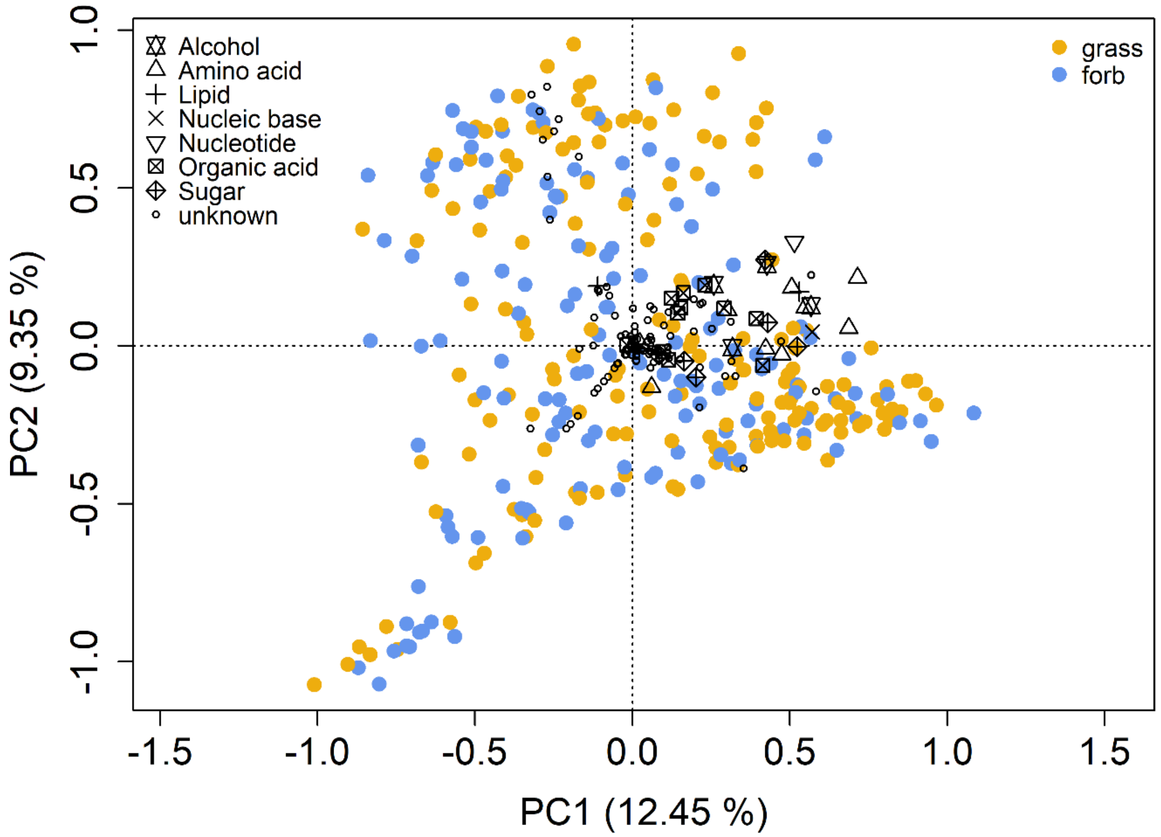


**S3 Fig. Principal component analysis (PCA) of polar metabolites.** Exudates were standardized by an internal standard (Ribitol (217)) and transformed into a presence/absence matrix. Colours represent the two growth forms. Symbols show compounds that could be attributed to the 7 substance classes (see S3 Table) and unknown compounds. This ordination was used to run a procrustes analysis (Fig. 3). To check for spatial autocorrelation we calculated Moran's I by using the scores of the first and second axis of the PCA as response variable and across all species found a marginal significant spatial autocorrelation on the first (observed difference in scores -0.008028366, expected -0.00330033, p = 0.091), but not on the second axis (observed difference in scores - 0.0007582542, expected - 0.00330033, p = 0.431). To exclude the plot effect, we calculated Moran's I for the first axis by species, which gave a marginal significant positive spatial autocorrelation for 1 out of the 10 species, *Ranunculus acris* (p=0.0501).
