# Supplementary material for: Linking root exudates to functional plant traits
Source: PLoS One. 2018 Oct 3;13(10):e0204128. doi: 10.1371/journal.pone.0204128 (PMC6169879; doi:10.1371/journal.pone.0204128)
Supplement: S1 File — (PDF) [file pone.0204128.s008.pdf]

**S1 File. Representative microscopic images of exuded plant roots stained by Trypan blue.** Dead and damaged root hair cells are coloured blue and marked by squares, whereas undamaged cells are colourless. Blue colour of the main root is provoked by the sum of staining of the cell layers.

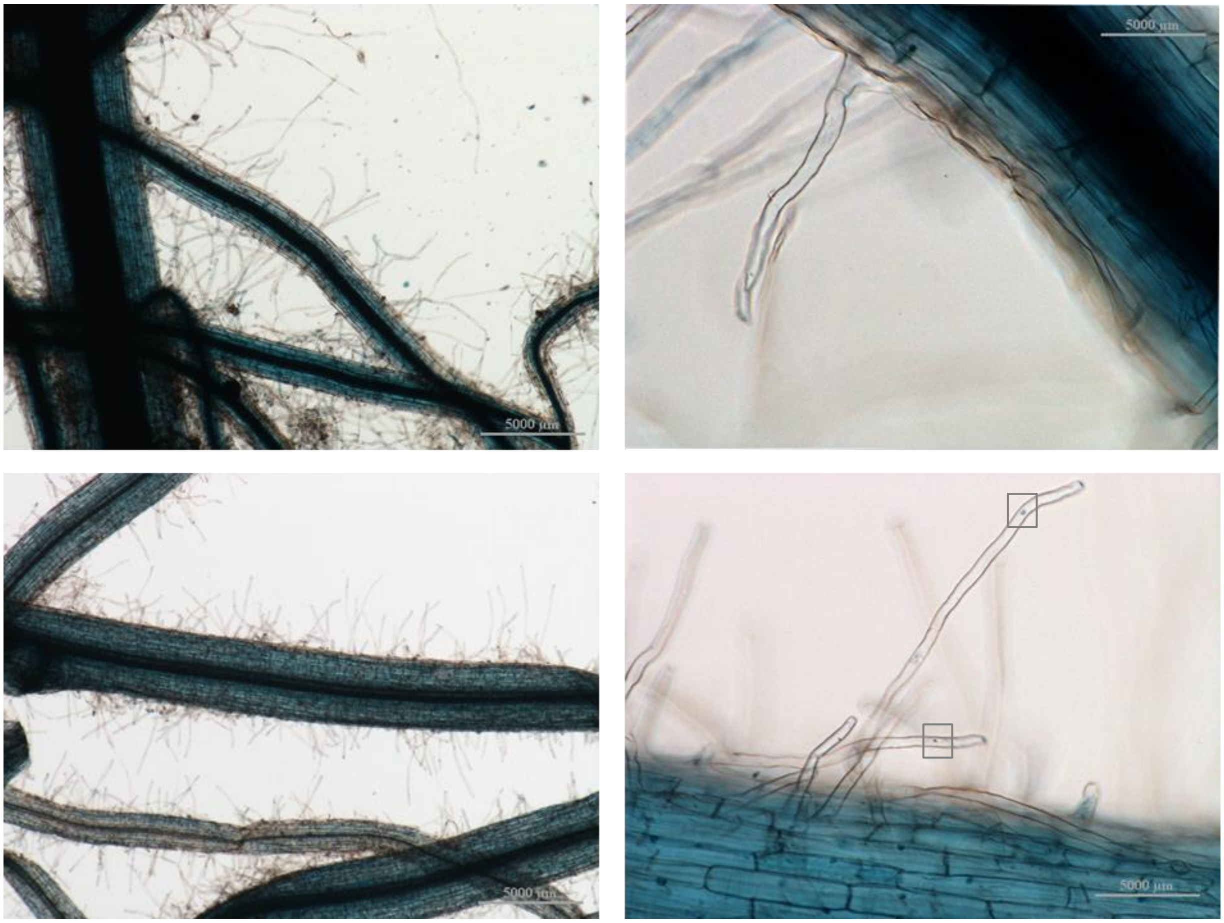

**a.** Top left: Overview image of *Plantago lanceolata* main root. Top right: Zoomed image of *P. lanceolata* root hairs. Bottom left: Overview image of *Arrhenatherum elatius* main root. Bottom right: Zoomed image of *A. elatius* root hairs.

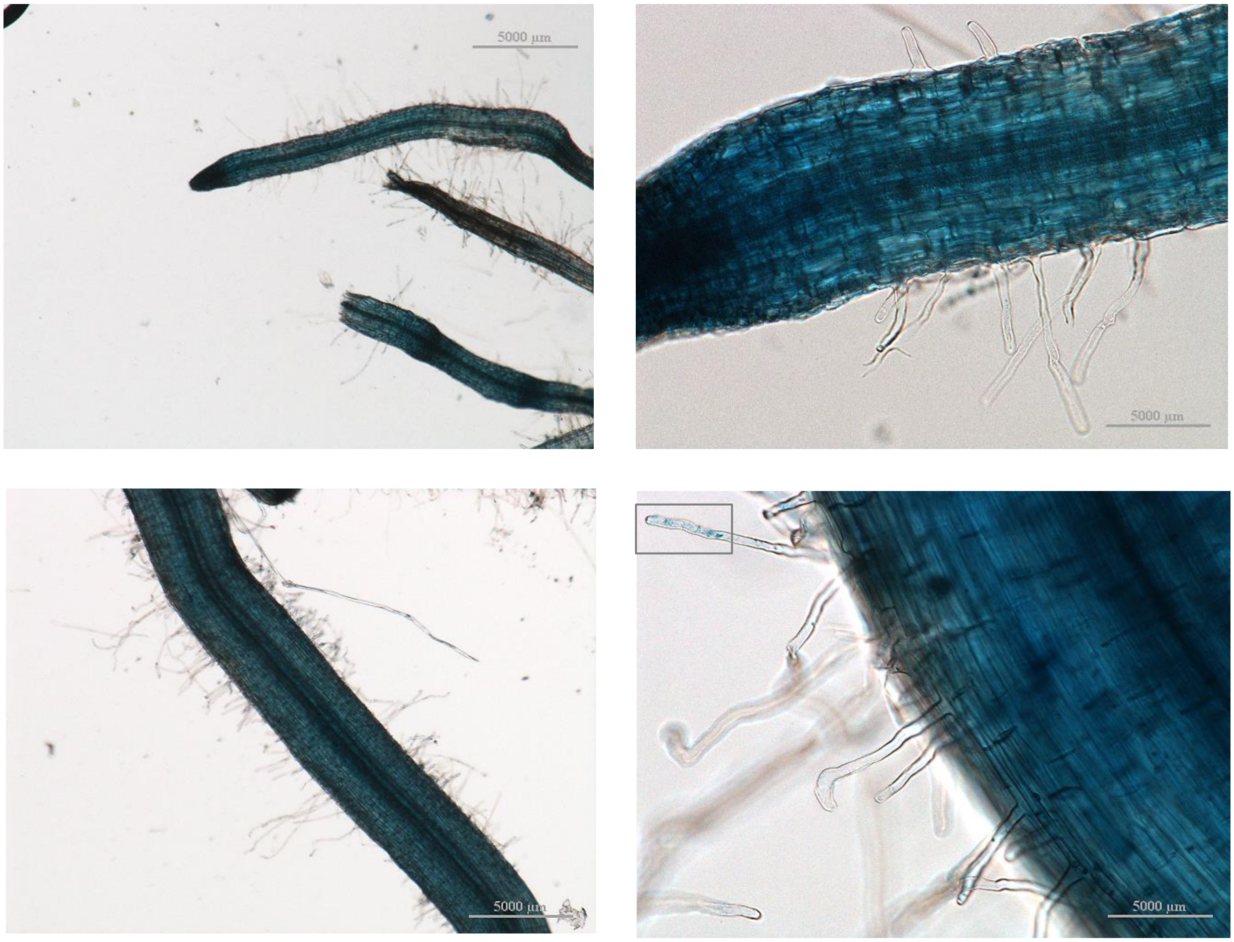


**b.** *P. lanceolata*: Top left: Overview image of *P. lanceolata* main root and root tip. Top right: Zoomed image of *P. lanceolata* root hairs at the root tip; bottom left: Overview image of *P. lanceolata* main root. Bottom right: Zoomed image of *Plantago lanceolata* root hairs

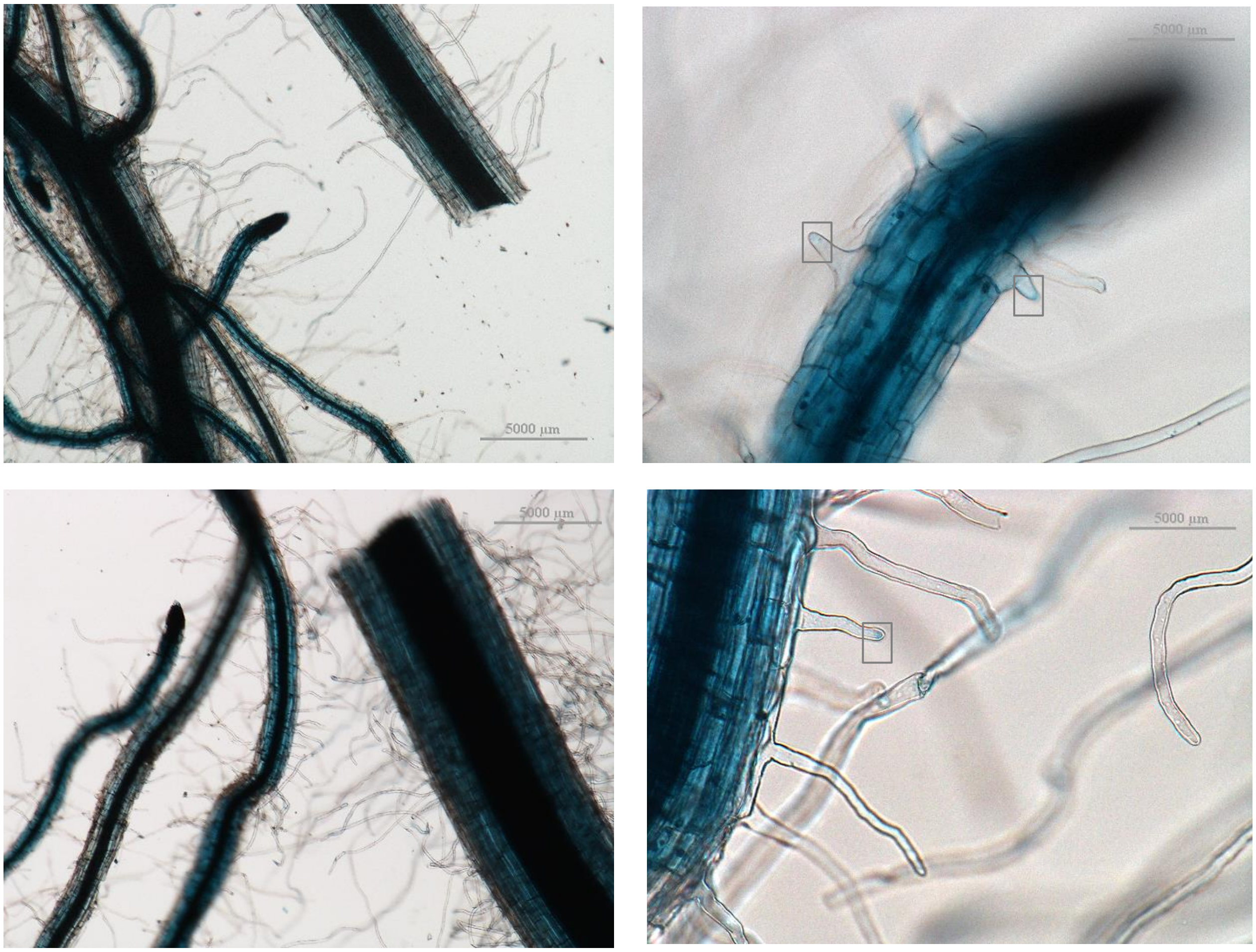


**c.** *A. elatius*: Top left: Overview image of *A. elatius* main root and root tip. Top right: Zoomed image of *A. elatius* root hairs at the root tip; bottom left: Overview image of *A. elatius* main root. Bottom right: Zoomed image of *A. elatius* root hairs
