# Supplementary material for: Linking root exudates to functional plant traits
Source: PLoS One. 2018 Oct 3;13(10):e0204128. doi: 10.1371/journal.pone.0204128 (PMC6169879; doi:10.1371/journal.pone.0204128)
Supplement: S2 File — (PDF) [file pone.0204128.s009.pdf]

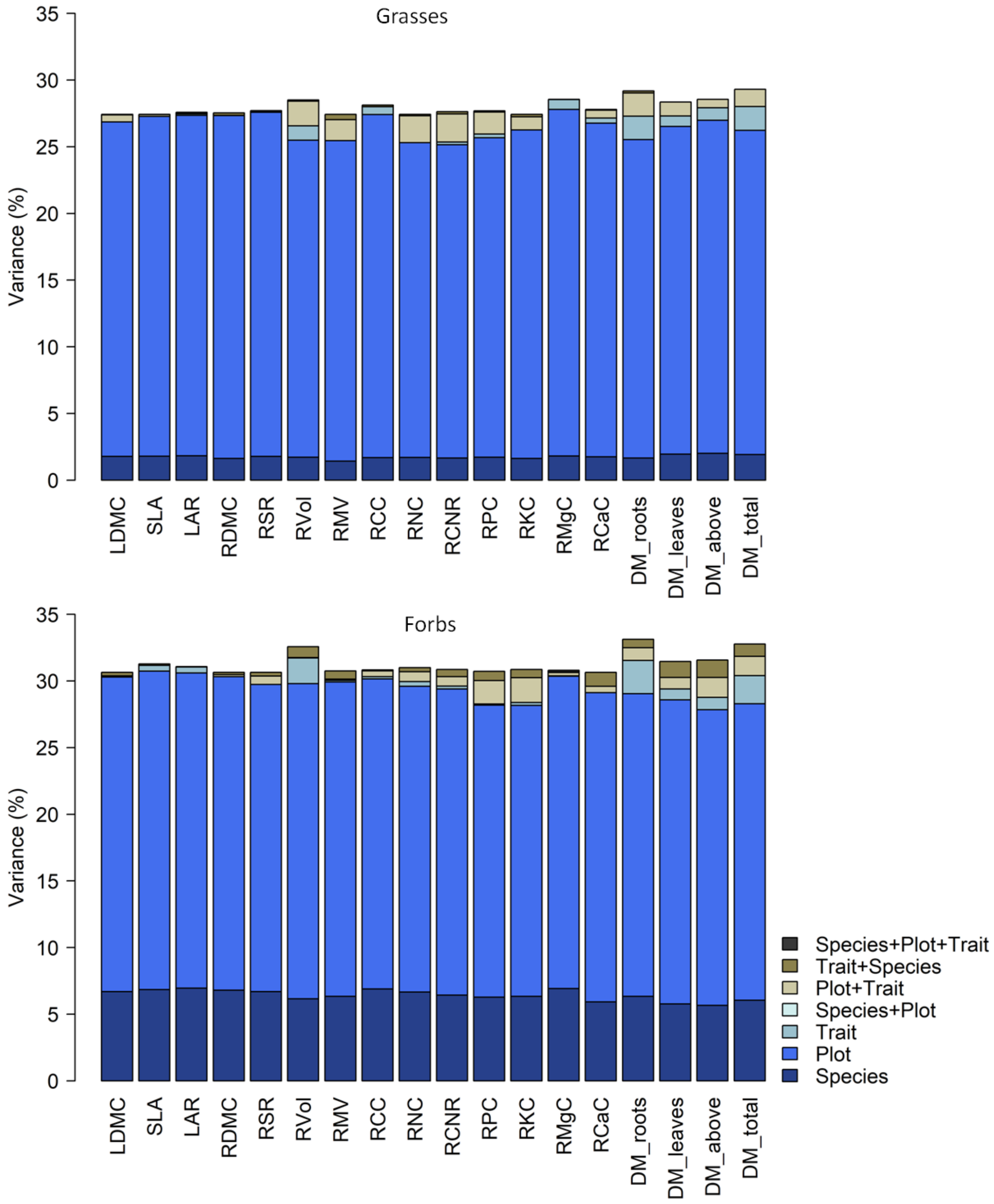

S2 File. Explained variance of exudate composition using single traits. We applied variance partitioning to a model containing target species identity, plot and one of the traits on the x-axis

as predictors separately for grasses and forbs. Residual variance is not shown. For abbreviations see S2 Table. For specific values see S3 File.
