# Supplementary material for: Linking root exudates to functional plant traits
Source: PLoS One. 2018 Oct 3;13(10):e0204128. doi: 10.1371/journal.pone.0204128 (PMC6169879; doi:10.1371/journal.pone.0204128)
Supplement: S3 File — (PDF) [file pone.0204128.s010.pdf]

S3 File. **Explained variance of exudate data in grasses and forbs using single traits.**

a. Explained variance of exudate data in grasses using single traits. The columns show exclusively explained variance by target species identity, plot and one of the listed traits, as well as the variance jointly explained by the combinations of these factors. For abbreviations see S2 Table.

	Species	Plot	Trait	Species+Plot	Plot+Trait	Trait+Species	Species+Plot+Trait	Residuals
LDMC	1.77	25.08	0.00	0.00	0.53	0.04	0.00	73.77
SLA	1.80	25.47	0.00	0.00	0.14	0.02	0.00	73.83
LAR	1.82	25.53	0.11	0.00	0.08	0.00	0.04	73.41
RDMC	1.63	25.70	0.03	0.00	0.00	0.18	0.00	73.49
RSR	1.78	25.81	0.03	0.00	0.00	0.04	0.05	73.48
Rvol	1.72	23.76	1.08	0.00	1.85	0.09	0.00	72.44
RMV	1.42	24.04	0.00	0.00	1.57	0.39	0.00	73.65
RCC	1.69	25.73	0.57	0.00	0.00	0.13	0.00	72.94
RNC	1.71	23.60	0.00	0.00	2.01	0.11	0.00	73.64
RCNR	1.65	23.49	0.20	0.00	2.12	0.16	0.00	73.32
RPC	1.72	23.96	0.27	0.00	1.65	0.09	0.00	73.25
RKC	1.63	24.63	0.00	0.00	0.98	0.19	0.00	73.51
RMgC	1.81	25.99	0.74	0.00	0.00	0.00	0.00	72.77
RCaC	1.75	25.03	0.38	0.00	0.58	0.07	0.00	73.14
DM_roots	1.66	23.87	1.76	0.00	1.74	0.16	0.00	71.75
DM_leaves	1.94	24.57	0.79	0.00	1.04	0.00	0.00	72.72
DM_above	2.01	24.97	0.93	0.00	0.64	0.00	0.01	72.58
DM_total	1.91	24.32	1.79	0.00	1.30	0.00	0.00	71.73

b. Explained variance of exudate data in forbs using single traits. The columns show exclusively explained variance by target species identity, plot and one of the listed traits, as well as the variance jointly explained by the combinations of these factors. For abbreviations see S2 Table.

	**Species**	**Plot**	**Trait**	**Species+Plot**	**Plot+Trait**	**Trait+Species**	**Species+Plot+Trait**	**Residuals**
LDMC	6.69	23.60	0.01	0.00	0.08	0.27	0.00	71.01
SLA	6.85	23.89	0.42	0.00	0.00	0.12	0.00	70.59
LAR	6.95	23.65	0.44	0.00	0.02	0.02	0.00	70.58
RDMC	6.81	23.51	0.00	0.00	0.17	0.16	0.00	71.26
RSR	6.69	23.05	0.00	0.00	0.63	0.28	0.00	71.04
Rvol	6.16	23.65	1.92	0.00	0.03	0.81	0.00	69.10
RMV	6.34	23.58	0.12	0.00	0.10	0.63	0.00	70.90
RCC	6.90	23.25	0.18	0.00	0.43	0.07	0.00	70.84
RNC	6.66	22.94	0.36	0.00	0.73	0.31	0.00	70.66
RCNR	6.43	22.97	0.21	0.00	0.71	0.54	0.00	70.81
RPC	6.27	21.92	0.08	0.00	1.76	0.70	0.00	70.94
RKC	6.34	21.83	0.22	0.00	1.85	0.63	0.00	70.80
RMgC	6.92	23.43	0.03	0.00	0.25	0.05	0.12	70.98
RCaC	5.92	23.21	0.00	0.00	0.47	1.05	0.00	71.15
DM_roots	6.34	22.71	2.48	0.00	0.97	0.62	0.00	68.54
DM_leaves	5.76	22.82	0.82	0.00	0.85	1.21	0.00	70.20
DM_above	5.67	22.18	0.92	0.00	1.50	1.30	0.00	70.10
DM_total	6.05	22.24	2.12	0.00	1.43	0.92	0.00	68.90
